# P-Glycoprotein–Related Interactions With Paliperidone May Result in Subtherapeutic Drug Levels and Clinical Exacerbation of Schizophrenia: A Case Report

**DOI:** 10.1097/JCP.0000000000002131

**Published:** 2026-01-26

**Authors:** Leonhard M. Kempinger, Florine M. Wiss, Markus L. Lampert, Adrian Sociu, Henriette E. Meyer zu Schwabedissen, Thorsten Mikoteit

**Affiliations:** 1 Biopharmacy, Department of Pharmaceutical Sciences, University of Basel, Basel, Switzerland h.meyerzuschwabedissen@unibas.ch; 2 Pharmaceutical Care, Department of Pharmaceutical Sciences, University of Basel, Basel, Switzerland; 3 Institute of Hospital Pharmacy, Solothurner Spitäler, Olten, Switzerland; 4 Psychiatric Services Solothurn Solothurner Spitäler and Department of Medicine, University of Basel, Solothurn, Switzerland

***To the Editors:***

Schizophrenia is a severe mental disorder with a global prevalence of ~23 million cases.^1^ Symptoms of schizophrenia are categorized as “positive” (hallucinations, delusions, and disorganized speech/behavior), “negative” (social withdrawal, anhedonia, and reduced emotional expression), and/or “cognitive” (impaired executive functioning and working memory).^2^ Treatment choice depends on multiple factors, including symptoms, comorbidities, and side-effect profile. Antipsychotics targeting the dopamine system are considered effective for positive symptoms, though they can also benefit other symptom domains.^3^ Negative and cognitive symptoms remain more difficult to treat and may require pharmacological strategies that modulate neurotransmitter systems beyond dopamine, including serotonin, glutamate, and acetylcholine.^4^ Nonetheless, variable treatment responses remain a significant challenge, with around 30% of patients experiencing treatment-resistant schizophrenia.^5^ One mechanism that causes interindividual differences in treatment response is the activity of drug-metabolizing enzymes and transporters, which is known to be affected by genetics, but also by pathological conditions (eg, diseases) and environmental factors (eg, diet or drug interactions).^6^ The latter factors might vary over time and could explain intraindividual variability in pharmacokinetics and efficacy of antipsychotics. In this context, we want to report specifically on 1 patient presenting with highly variable paliperidone serum concentrations.

## CASE REPORT

The 49-year-old male patient was diagnosed with schizophrenia (International Classification of Diseases 10th Revision, ICD-10: F20.0) and posttraumatic stress disorder (ICD-10: F43.1) with dissociative symptoms, and a persistent personality change following extreme stress (ICD-10: F62.0). Nonpsychiatric diagnoses comprised trigeminal neuralgia (ICD-10: G50.0) and enuresis of nonorganic origin (ICD-10: F98.0). In detail, the patient experienced multimodal hallucinations—auditory, visual, and tactile—accompanied by intense anxiety, including a strong sense of impending death. He also expressed a specific and persistent fear of vampires coupled with the delusional belief that he was transforming into one, perceiving himself as a potential threat to both himself and his family.

The patient was a heavy smoker, and he suffered from severe sleep disturbance due to pronounced restlessness and anxiety. Organic psychosis has been ruled out by blood tests and drug screening, while neuroimaging has excluded any neurostructural backgrounds. He had a long-standing history of schizophrenia with recurrent hospitalizations due to psychotic exacerbations often involving self-directed or outward aggression, despite receiving long-acting injectable antipsychotics to improve adherence and maintain therapeutic efficacy through more stable serum concentrations. Since late 2017, the patient received monthly long-acting injectable paliperidone (150 mg), which led to paliperidone serum concentrations within the therapeutic reference range (TRR; 50 to 150 nmol/L).

We want to highlight 2 episodes within the patient’s medical history, the first in early 2024. Here, the patient was rehospitalized due to an exacerbation of psychotic symptoms. The measured paliperidone serum concentration was 57 nmol/L, placing it at the lower end of the TRR. In the hospital, his treatment with carbamazepine (up to 800 mg daily) was discontinued due to a drug-drug interaction with paliperidone. In addition, paliperidone was switched from the monthly (150 mg) to the 3-monthly formulation (525 mg), under which the patient clinically improved and stabilized.

The second episode began at the end of 2024, where the patient presented for voluntary admission due to psychotic symptoms with a measured paliperidone concentration of 43 nmol/L. This finding was unexpected, as the most recent injection had been administered in late 2024, with the next dose not scheduled before early 2025. In response to the subtherapeutic drug levels, oral paliperidone (3-6 mg/d) was combined with the readministration of the injectable formulation. To further optimize antipsychotic coverage, the patient’s quetiapine dose was increased in 2 steps to 200 mg (oral) daily. The patient additionally received 15 mg of aripiprazole daily for the management of persistent psychotic symptoms. Despite this multi-antipsychotic approach and restoration of therapeutic paliperidone serum levels (95 to 126 nmol/L), only partial remission of symptoms was achieved, necessitating a 1-week extension of hospital care. Because of the inexplicably low serum concentration of paliperidone, the hospital’s clinical pharmacy service was consulted to review the patient’s medication regimen for potential contributing factors. It was noted that the patient was consuming exceptionally large quantities of garlic (up to 3 heads per day), reportedly as a protective measure against vampires, which was his delusional concept. The clinical pharmacists suspected a possible drug-food interaction between paliperidone and garlic, and recommended discontinuation of garlic intake. In addition, they advised considering maintaining oral supplementation of paliperidone to support therapeutic efficacy during long-acting injectable treatment. At discharge, the patient had reduced his garlic intake to 2 heads per day, but treatment with oral paliperidone (3 mg/d) in addition to the injectable formulation was continued. With 2 cloves of garlic daily, at a paliperidone serum concentration of 178 nmol/L, the patient achieved a stable psychiatric state. He gave written informed consent for this case to be published.

## DISCUSSION

In this case report, exacerbation of chronic schizophrenia coincided with a decrease in paliperidone concentrations despite regular injections of long-acting paliperidone. This atypical antipsychotic is available in both oral and parenteral formulations. Because of its low oral bioavailability, it is most commonly administered as a long-acting injectable. Pharmacologically, paliperidone is the major active metabolite of risperidone. But in contrast to risperidone, paliperidone is predominantly excreted unchanged renally and shows minimal hepatic metabolism,^7^ making it less susceptible to drug-drug interactions. However, there is evidence that paliperidone is a substrate of P-glycoprotein (P-gp).^8^ This ATP-dependent efflux pump is expressed in tissues with pharmacokinetic relevance, such as the endothelial cells of the blood-brain barrier, the intestinal epithelium, the hepatocytes in the liver, and the epithelial cells of the renal proximal tubules. Accordingly, P-gp is assumed to play a critical role in pharmacokinetics by limiting drug absorption and enhancing drug elimination into the bile or the urine.

In the context of this case, we want to discuss 2 possible mechanisms of P-gp–related decline in paliperidone serum concentrations: First, a possible drug-drug interaction, second, a drug-food interaction, both of which result in clinically relevant symptom exacerbation. The first occasion of an unexpected decline in paliperidone serum concentrations was in early 2024. At that time, the patient was treated with intramuscular injections of paliperidone, oral quetiapine, and carbamazepine. The latter is an anticonvulsant and was administered for the treatment of trigeminal neuralgia. The reduction of the patient’s paliperidone serum levels is in line with the previously reported reduced paliperidone plasma levels in 6 patients who were administered increasing doses of carbamazepine together with oral paliperidone (6 to 12 mg/d).^9^ The interaction between paliperidone and carbamazepine is most likely linked to the fact that carbamazepine is an activator of the nuclear receptor PXR, which is a ligand-activated transcription factor responsible for the induction of several genes involved in drug metabolism and transport, such as CYP3A4 and P-gp.^10^ Although PXR-mediated metabolic induction is primarily considered relevant in the context of hepatic drug metabolism and transport, it is important to note that PXR is also expressed in other tissues, including the intestine and kidneys, where it can similarly influence drug disposition.^11^ Induction of intestinal P-gp expression and function has been clearly linked to reduced oral bioavailability of the P-gp substrate digoxin, however upon treatment with rifampicin.^12^ The same mechanism may have also contributed to the observed reduction of paliperidone plasma levels in the 6 orally treated patients.^9^ However, Kerbusch-Herben et al^13^ suggested a limited impact of carbamazepine on the oral bioavailability of paliperidone while describing a clear increase in renal clearance, based on their findings in 64 patients. Importantly, changes in renal clearance following P-gp induction have also been reported after parenteral administration of the P-gp probe drug talinolol.^14^ In the presented case, the reduction in serum levels following parenteral administration of paliperidone may therefore be due to carbamazepine-induced increased renal clearance. Considering that the patient stabilized after discontinuation of carbamazepine supports the relevance of the above-discussed mechanisms in the handling of paliperidone, even if not orally administered. Concerning a possible contribution of quetiapine to the P-gp–dependent paliperidone levels, the evidence is inconsistent, because quetiapine is both an activator of PXR,^15^ but can also act as an inhibitor of P-gp.^16^ In this context, it should be mentioned that quetiapine is a substrate of CYP3A4, a drug-metabolizing enzyme, which is also induced upon PXR activation.^17^

For the second decline of paliperidone serum levels, induction of P-gp might have played a role, though not due to carbamazepine co-administration. The last exacerbation of psychotic symptoms coincided with an excessive consumption of 2 to 3 heads of garlic per day. Clinical data on drug-food interactions involving garlic, garlic extracts, and/or supplements are rather limited and conflicting.^18^ Yet, some human studies clearly demonstrate intestinal P-gp induction following treatment with a garlic extract.^19^ Well-known constituents of garlic are organosulfur compounds such as allyl sulfide/trisulfides, diallyl disulfides, among which allyl disulfides have been shown to activate CAR at least in rats.^20^ This nuclear receptor shares several target genes with PXR, including P-gp. Nonetheless, there is no in vitro evidence supporting that these constituents are also ligands and activators of human PXR or CAR, which would support a potential drug-food interaction involving P-gp. The improvement in the patient’s symptoms, along with the normalization of serum concentration following the reduced garlic consumption, led us to consider that garlic may have contributed to the shift in his pharmacokinetic balance. In addition, the shorter injection intervals, the supplementation with oral paliperidone, and the concomitant increase in oral quetiapine dosing, a P-gp inhibitor, may have played an important role in the normalization of paliperidone serum levels. Finally, P-gp is relevant for the blood-brain barrier functionality. In this regard, we want to state that it is currently not known whether the transporter’s expression and activity in brain capillaries are also affected by PXR in humans, which may have contributed to the exacerbation of the symptoms independent of serum levels.

In conclusion, we present a biopharmaceutical mechanistic interpretation of the patient’s medical history with recurrent psychotic relapses and subtherapeutic paliperidone serum levels, despite regular administration of long-acting paliperidone. It is our intention to emphasize the need for a more comprehensive and nuanced approach to antipsychotic management. This case illustrates that environmental factors, including drug-drug and drug-food interactions, are likely to underlie intraindividual variability in treatment outcomes—even for parenterally administered medications, which are primarily renally eliminated.
